# Factors influencing intrapatient variability of tacrolimus and its association with 1-year post-transplant outcomes in pediatric liver transplant recipients

**DOI:** 10.3389/fphar.2024.1473891

**Published:** 2024-11-21

**Authors:** Chuxuan Fang, Chunqiang Dong, Kaiyong Huang, Ningyu Wen, Yiyu Chen, Shuangyi Tang

**Affiliations:** ^1^ Department of Pharmacy, The First Affiliated Hospital of Guangxi Medical University, Nanning, China; ^2^ Department of Organ Transplantation, The First Affiliated Hospital of Guangxi Medical University, Nanning, China

**Keywords:** child, liver transplantation, tacrolimus, intrapatient variability, coefficient of variation

## Abstract

**Objective:**

This study aims to explore the factors influencing tacrolimus intrapatient variability (TAC-IPV) and its association with 1-year post-transplant outcomes in pediatric liver transplant recipients.

**Methods:**

Clinical and biological data of pediatric patients after liver transplantation were collected. The patients were divided into high- and low-IPV groups according to the median TAC-IPV for statistical comparisons. Factors with *p* < 0.05 in univariate analysis were introduced into binomial logistic regression analysis. Correlation analysis was used to test the connections between the Tac-IPV and outcomes within 1 year after liver transplantation (LT), and Kaplan–Meier was used to draw the survival curves.

**Results:**

A total of 116 children underwent 746 measurements of TAC trough concentrations. The median TAC-IPV was 32.31% (20.81%, 46.77%). Hematocrit (*p* = 0.017) and concomitant medications (*p* = 0.001) were identified as independent influencing factors for TAC-IPV. The incidence of transplant rejection (*p* = 0.008), CMV infection (*p* < 0.001), and hospital admission due to infection (*p* = 0.003) were significantly higher in the high-IPV group than in the low-IPV group. Kaplan–Meier survival analysis suggests that after considering the time factor, high IPV (IPV > 32.31%) was still significantly associated with transplant rejection (HR = 3.17 and *p* = 0.005) and CMV infection (HR = 2.3 and *p* < 0.001) within 1 year after LT.

**Conclusion:**

The study highlights the significant variation in TAC-IPV among children post-liver transplantation, emphasizing the impact of hematocrit levels and concomitant medications on TAC-IPV. Elevated TAC-IPV is associated with increased risks of transplant rejection, CMV infection, and readmission due to infection in the first year after liver transplantation. Close monitoring of patients with high TAC-IPV is recommended to promptly detect adverse reactions and provide timely intervention and treatment.

## 1 Introduction

Liver transplantation (LT) is an effective method for treating end-stage liver diseases and genetic metabolic diseases in children (Spada et al., 2009). The common causes of pediatric LT in clinic include primary biliary atresia, hepatoblastoma, and metabolic disease. Since the first pediatric LT in 1963, with the innovation of LT technologies, the standardization of multidisciplinary perioperative management, the development of immunosuppressants, and the experience accumulation of long-term management and follow-up, pediatric LT has developed rapidly, and the post-operative survival rate and long-term quality of life of children have significantly improved (He et al., 2024; Kokudo et al., 2020). After LT, patients generally typically require long-term immunosuppressants to prevent acute and chronic transplant rejection. Commonly used medications for solid organ transplantation mainly include calcineurin inhibitors (CNIs), mycophenolate mofetil, sirolimus, corticosteroids, and induction therapy agents. In Europe, the United States, and Asia, CNIs are recommended as the principal choice for immunosuppression after LT, with tacrolimus (TAC) being the preferred drug for the majority of liver transplant recipients (2016, Kim et al., 2024).

Tacrolimus, a macrolides CNI isolated from the fermentation broth of *Streptomyces tsukubaensis*, exerts its effects on the activation and proliferation of T cells by inhibiting the transcription of the *interleukin-2* gene (Starzl et al., 1989; Liu et al., 2024). Since its introduction into clinical practice in the early 1990s, it has been widely utilized for the prevention of rejection after transplantation (Brunet et al., 2019; Scalea et al., 2016). However, the therapeutic window of TAC is narrow, and there are substantial inter-individual and intra-individual differences in pharmacokinetic characteristics (Shuker et al., 2016). This is particularly challenging in pediatric LT patients as the changes in various aspects, such as physiology, biochemistry, and maturational physiology, along with the pharmacokinetic profile of drugs, are more complex, making the use and dosage adjustment of TAC more difficult. A study has revealed that under the standard dose, over 50% of pediatric LT patients may suffer acute rejection, nephrotoxicity, neurotoxicity, or other adverse reactions (Musuamba et al., 2014).

Intrapatient variability (IPV) is defined as the fluctuation of trough TAC concentrations over a period of time under the condition of a stable drug dose, which describes the variation in TAC trough concentrations over time (Shuker et al., 2015). TAC-IPV provides a better assessment of the risk of adverse outcomes caused by TAC’s rapid fluctuations in concentration between over- and underexposure. Commonly utilized indicators for calculating or representing TAC-IPV include standard deviation (SD), coefficient of variation (CV), TAC variability score (TVS), and time-weighted CV (Barbora et al., 2022).

At present, research on TAC-IPV after LT mainly focuses on adults, with only a few studies addressing the relevant content of TAC-IPV in children after LT. This study aims to investigate the relationship between TAC-IPV and post-transplantation graft rejection, Epstein–Barr virus (EBV) infection, cytomegalovirus (CMV) infection, and hospital readmission due to infection in pediatric patients. Furthermore, the study seeks to identify the factors that influence TAC-IPV in this patient population.

## 2 Materials and methods

### 2.1 Patient population

This retrospective study included pediatric patients who underwent LT and received TAC at the First Affiliated Hospital of Guangxi Medical University from July 2017 to December 2022. Ethical approval for this study (approval number: 2024-E455-01) was provided by the Medical Ethics Committee of the First Affiliated Hospital of Guangxi Medical University on 21 June 2024. This study was conducted in accordance with both the Declarations of Helsinki and Istanbul.

The inclusion criteria were as follows: patients aged <18 years, undergoing initial liver transplantation, with a follow-up period >1 year, and at least three times TAC trough concentrations taken on the day of admission or outpatient reviews during 4–9 months after LT. Additionally, the clinical and biological data must be complete. The exclusion criteria were included multiple organ transplantation and secondary liver transplantation within 1 year after surgery.

### 2.2 Immunosuppressive protocol

A combination immunosuppressive protocol of TAC, mycophenolate mofetil, and steroid was used. The initial dose of TAC was 0.05 mg/kg, which was then adjusted according to the TAC trough concentrations to achieve the target range: 10–12 ng/mL in the first month, 8–10 ng/mL for 2–3 months, and 6–8 ng/mL for 4–12 months. TAC trough concentrations were monitored monthly during follow-up.

### 2.3 TAC detection method

Whole blood concentrations of TAC were detected using an electrochemiluminescent immunoassay (cobas e 801). Sample preparation was performed according to the reagent instructions: 300 μL of the whole blood sample was transferred to a centrifuge tube, followed by the addition of 300 μL of sample extraction liquid. The mixture was vortexed for 10 s and then centrifuged for 4 min. The supernatant was collected for determination.

### 2.4 Patient data collection and definition

Clinical and biological data were retrospectively retrieved from the Hospital Information System, including gender, nationality, age, weight, and type of donor. Biological data included total bilirubin, aspartate transaminase (AST), alanine aminotransferase (ALT), creatinine, creatinine clearance rate (CCR), white blood cell count (WBC), red blood cell count (RBC), hemoglobin, platelet, hematocrit (HCT), prothrombin time (PT), fibrinogen (FIB), activated partial thromboplastin time (APTT), and clotting time (TT). Follow-up data included TAC trough concentrations on the day of admission or during outpatient reviews and concomitant medications taken during 4–9 months after LT; additional data included post-transplantation graft rejection, new-onset diabetes and hypertension, EBV and CMV infections, and other infections within 1 year after LT. The information regarding rehospitalization for infection included the time, site and species of infection, and relevant test results.

### 2.5 Intrapatient tacrolimus variability and other definitions

IPV was expressed as the coefficient of variation (CV) of individual TAC trough concentrations. The formula for calculating CV is as follows: σ represents the SD of TAC concentrations per patient, and μ represents the average of TAC concentrations per patient. The IPV value for each patient was calculated individually based on the measured TAC trough concentrations, and then the median of TAC-IPV of the entire cohort was used as the cut-off to divide the patients into low- and high-IPV groups.

$$CV=\frac{\sigma }{\mu }\times 100\%.$$

CMV/EBV infection was defined as CMV/EBV replication in blood (detected by PCR), regardless of symptomatology. CMV replication was identified using quantitative nucleic acid testing, with a positive result indicated by nucleic acid quantification greater than 400. Similarly, the EBV DNA load was measured using fluorescence quantitative PCR, with a positive result defined as a DNA copy number exceeding 400.

### 2.6 Statistical analysis

Stata/MP 18.0 and GraphPad Prism 8.3.0 were used for statistical analysis. Normally distributed continuous variables were expressed as the mean ± SD and compared using the independent sample t-tests. Non-normally distributed continuous variables were expressed as the median (quartiles), and the Mann-Whitney *U*-test was used for comparison. Qualitative variables were expressed as numbers and percentages, with comparisons between groups carried out using the Pearson chi-square or Fisher’s exact tests. Binary logistic regression included variables with a *p* < 0.05 from the univariable analysis. Survival was assessed using the Kaplan–Meier curves and compared using the log-rank test. *p* < 0.05 value was considered statistically significant.

## 3 Results

### 3.1 General characteristics of pediatric LT patients

A total of 116 pediatric patients who underwent LT and met the inclusion criteria were included in this study. The patients ranged in age from 3 to 188 months, with a median age of 9 (6, 36.75) months. The majority of patients were male (67 cases, 59.48%). A total of 746 TAC trough concentrations were collected. The TAC-IPV was calculated for each patient, and the median TAC-IPV was 32.31%, which was used as the cut-off. The IPV difference between the two groups was 25.96% (20.81% vs. 46.77%). A total of 341 TAC trough concentrations were obtained in the low-IPV group, with an overall mean of 6.93 (patients’ mean trough concentrations ranged from 3.27 to 12.63 ng/mL). In the high-IPV group was 405 TAC trough concentrations were collected, with a mean of 7.18 ng/mL (patients’ mean trough concentrations ranged from 2.97 to 12.8 ng/mL). We found that 61.88% of trough concentrations in the low-IPV group and 82.24% in the high-IPV group were outside the target range, while 25.81% and 31.60% of these groups, respectively, were above the target range. The basic diseases and other detailed information of the pediatric patients are shown in Table 1. There was no significant difference in mean TAC trough concentration or dosing between the two groups.

**TABLE 1:** Results of general information and univariate analysis between the two groups.

Factor	Low IPV (n = 58)	High IPV(n = 58)	x ^2^/*Z*/*t*	*p*
Mean concentration, ng/mL	6.93 ± 1.85	7.18 ± 2.51	−0.607	0.545
Mean daily dosage, mg	3.10 ± 1.46	2.87 ± 1.45	0.928	0.355
Gender			1.753	0.186
Male, n (%)	38 (65.52)	31 (53.45)		
Female, n (%)	20 (34.48)	27 (46.55)		
Nation			0.552	0.457
The Han nationality, n (%)	32 (55.17)	28 (48.28)		
National minority, n (%)	26 (44.83)	30 (51.72)		
Blood type			1.555	0.694
O, n (%)	26 (44.83)	20 (34.48)		
A, n (%)	11 (18.97)	13 (22.42)		
B, n (%)	18 (31.03)	20 (34.48)		
AB, n (%)	3 (5.17)	5 (8.62)		
Age, M	9.00 (6.00,44.00)	9.00 (6.00,29.00)	−0.014	0.989
Height, cm	68.00 (64.00,94.00)	68.50 (63.00,81.00)	0.108	0.914
Weight, kg	8.00 (6.75,14.00)	7.60 (6.40,11.00)	0.260	0.795
BMI, kg·m^-2^	16.09 (14.78,17.01)	15.93 (14.92,16.99)	0.295	0.768
Body surface area, m^2^	0.37 (0.32,0.61)	0.36 (0.32,0.48)	−0.235	0.814
Primary disease			0.470	0.791
Cholestatic liver disease, n (%)	45 (77.58)	44 (75.86)		
Inherited metabolic diseases/n (%)	4 (6.90)	6 (10.35)		
Other diseases/n (%)	9 (15.52)	8 (13.79)		
Donor type			0.719	0.396
Living donor LT, n (%)	41 (70.69)	45 (77.59)		
Cadaveric donor LT, n (%)	17 (29.31)	13 (22.41)		
Anemia			1.052	0.305
Y, n (%)	7 (12.07)	11 (18.97)		
N, n (%)	51 (87.93)	47 (81.03)		
Hypoproteinemia			3.013	0.083
Y, n (%)	18 (31.03)	10 (17.24)		
N, n (%)	40 (68.97)	48 (82.76)		
Medicine combination			11.226	0.001
Y, n (%)	22 (37.93)	40 (68.97)		
N, n (%)	36 (62.07)	18 (31.03)		
Total bilirubin, μmol/L	40.60 (17.30,69.80)	45.65 (18.40,75.00)	−0.425	0.671
AST, U/L	77.00 (42.00,277.00)	153.50 (53.00,364.00)	−2.040	0.041
ALT, U/L	192.00 (96.00,385.00)	252.00 (149.00,466.00)	−1.311	0.190
Creatinine, μmol/L	15.50 (12.00,23.00)	16.00 (13.00,23.00)	−0.395	0.693
CCR, mL/min	86.35 (76.80,101.00)	99.50 (77.00,118.70)	−1.715	0.086
WBC, 10^9/L	9.11 (5.98,13.17)	11.00 (6.74,14.51)	−1.391	0.164
RBC, 10^12/L	3.49 ± 0.71	3.70 ± 0.67	−1.683	0.095
Hemoglobin, g/L	94.80 (87.20,104.00)	101.45 (91.80,114.20)	−2.341	0.019
Platelet, 10^9/L	121.30 (78.00,188.80)	124.20 (83.90,196.00)	0.110	0.912
Hematocrit, %	28.79 ± 4.65	31.15 ± 5.28	−2.547	0.012
PT, s	16.35 (14.40,20.50)	15.95 (14.10,20.70)	0.097	0.923
FIB, g/L	1.76 (1.30,2.20)	1.81 (1.32,2.44)	−0.776	0.438
APTT, s	46.05 (40.10,53.30)	46.85 (39.20,60.10)	−0.375	0.707
TT, s	14.35 (11.80,22.10)	14.60 (11.00,20.60)	0.373	0.709

n, number; M, month; BMI, body mass index; LT, liver transplantation; Y, yes; N, no; AST, aspartate transaminase; ALT, alanine aminotransferase; CCR, creatinine clearance rate; WBC, white blood cell count; RBC, red blood cell count; PT, prothrombin time; FIB, fibrinogen; APTT, activated partial thromboplastin time; TT, clotting time; IPV, intrapatient variability.

### 3.2 Analysis of influencing factors of TAC-IPV

In the univariate regression analysis, variables such as AST (*p* = 0.041), hemoglobin (*p* = 0.019), HCT (*p* = 0.012) before TAC use, and medicine combination (*p* = 0.001) were related to the TAC-IPV, as shown in Table 1. The results of binary logistic regression with stepwise regression showed that HCT (*p* = 0.017) and medicine combination (*p* = 0.001) were independent risk factors for high-IPV in children after LT, as shown in Table 2.

**TABLE 2:** Binary logistic regression analysis of influencing factors of TAC-IPV in children after liver transplantation.

	*β*	*SE*	*z*	*p*	*OR*	95% *CI*
Hematocrit, %	0.102	0.043	2.39	0.017	1.1076	1.0187~1.2042
Medicine combination	1.318	0.405	3.26	0.001	3.7368	1.6901~8.2620
Constant	−3.760	1.327	−2.83	0.005	0.0233	0.0017~0.3137

### 3.3 Association of TAC-IPV with post-transplant outcomes within 1 year

Within 1 year after pediatric LT, there were three cases of new-onset diabetes (2.59%) and three cases of new hypertension (2.59%), one of which progressed to hypertensive encephalopathy (IPV, 60.58%). Graft-versus-host disease (GVHD) was observed in three patients at 5, 6, and 10 months, with IPV values of 33.60%, 37.33%, and 53.29%, respectively. One patient was diagnosed with post-transplant lymphoproliferative disorder (PTLD) and diffuse large B-cell lymphoma at 5 months after LT (IPV, 41.97%). Three patients died: one due to severe steroid-dependent refractory rejection combined with sepsis and pneumonia; and two died from acute respiratory distress syndrome (ARDS) resulting from severe pneumonia caused by bacteria, fungi, and viruses, with one of these patients infected with multi-drug resistant *Acinetobacter baumannii*. The IPV values for these patients were 33.60%, 31.11%, and 45.28%, respectively. The incidence of transplant rejection (*p* = 0.008), CMV infection (*p* < 0.001), and hospital admission due to infection (*p* = 0.003) in the high-IPV group were significantly higher than those in the low-IPV group. There was no statistical difference between the two groups regarding anemia, hepatitis B virus (HBV) infection, and EBV infection. Details are shown in Table 3.

**TABLE 3:** Complications and adverse reactions within 1 year after liver transplantation in children, cases (%).

Factor	Low IPV (n = 58)	High IPV (n = 58)	x ^2^	*p*
Diabetes	0	3 (5.17)		
Hypertension	1 (1.73)	2 (3.45)		
Anemia	11 (18.97)	17 (29.31)	1.695	0.193
GVHD	0	3 (5.17)		
Post-transplantation graft rejection	7 (12.07)	19 (32.76)	7.138	0.008
HBV infection	2 (3.45)	6 (10.34)	2.148	0.272
EBV infection	28 (48.28)	31 (53.45)	0.310	0.577
CMV infection	25 (43.10)	44 (75.86)	12.913	0.000
Times of hospital readmission due to infection			11.658	0.003
0	33 (56.90)	15 (25.86)		
1~2	18 (31.03)	29 (50.00)		
≥3	7 (12.07)	14 (24.14)		

GVHD, graft-versus-host disease; HBV, hepatitis B virus; EBV, Epstein–Barr virus; CMV, cytomegalovirus; n, number.

**FIGURE 1:**
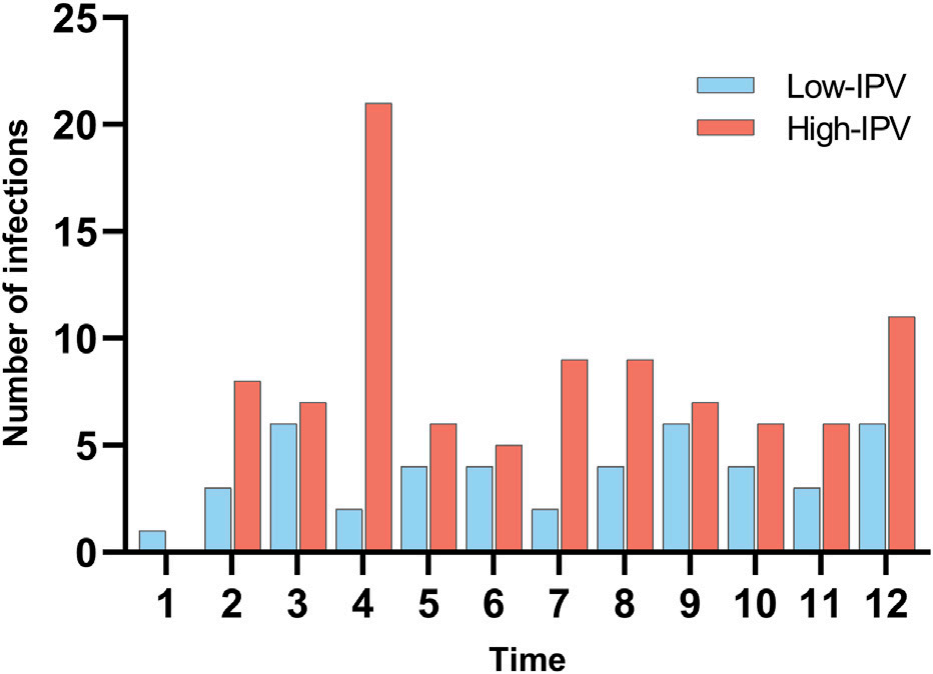
Time distribution of readmissions due to infection.

In this study, a total of 140 readmissions to hospital due to infection within 1 year after pediatric LT were collected. Among them, respiratory system infections accounted for 54.29%, followed by digestive system infections at 20.71%. In the microbial culture of identified pathogens, except EBV and CMV, the top five pathogens were *Candida albicans* (five cases), *Candida glabrata* (four cases), *Candida tropicalis* (three cases), rotavirus (three cases), and adenovirus (three cases). In addition, five types of multi-drug resistant bacteria were detected, namely, *Klebsiella aerogenes* (IPV 45.67%), *Pseudomonas aeruginosa* (IPV 55.17%), *Escherichia coli* (IPV 45.67%), *Staphylococcus aureus* (IPV 41.11%), and *A. baumannii* (IPV 31.11%). Figure 1 illustrates the timing and frequency of infections across different IPV groups.

### 3.4 Kaplan–Meier survival analysis

**FIGURE 2:**
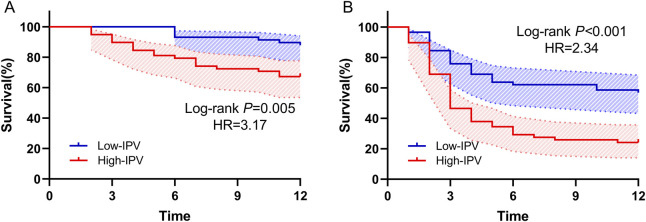
Kaplan–Meier analysis of transplant rejection and CMV infection in high- and low-IPV groups. **(A)** Transplant rejection and **(B)** CMV infection.

Patients were divided into low-IPV and high-IPV groups based on median TAC-IPV (32.31%). Transplant rejection and CMV infection were taken as outcome events, respectively, as shown in Figure 2. Kaplan–Meier analysis revealed a strong association between high IPV and an increased risk of transplant rejection (HR = 3.17 [1.47; 6.86]; *p* = 0.005) and CMV infection (HR = 2.34 [1.45; 3.76]; *p* < 0.001) within 1 year. This indicates that patients with IPV above 32.31% had a significantly higher hazard of experiencing transplant rejection and CMV infection.

## 4 Discussion

### 4.1 Background and study design

In recent years, TAC-IPV has become a novel potential biomarker in the management of solid organ transplant recipients. In kidney transplantation, several studies suggest that high IPV is related to poorer outcomes (Kim et al., 2021; Kim et al., 2023; Xie et al., 2024; Larpparisuth et al., 2021; Mendoza Rojas et al., 2022). In pediatric LT, only a few studies explored the relationship between TAC-IPV and clinical outcomes. Previous studies have found that high IPV is associated with acute rejection, recipient survival, graft survival, and the need for biopsy during the 1st year (Riva et al., 2018; Chen et al., 2024; Defrancq et al., 2019), but the studies on CMV and EBV infections are still lacking. In order to prevent graft rejection, pediatric patients usually need adjustments in immunosuppressive treatments, which may lead to an increased incidence of various opportunistic infections (including CMV, EBV, and invasive fungal infections) (Abdullatif et al., 2023; Pana et al., 2017; Farahani et al., 2023). Due to the heterogeneity of protocols in the management of CMV surveillance and antiviral prophylaxis, the incidence of CMV infection after LT in children ranges from 15% to 70% (Abdullatif et al., 2023). Studies have shown that CMV infection is associated with an increased risk of graft loss, invasive fungal disease, and long-term mortality, posing a great threat to post-operative survival (Kleiboeker et al., 2022; Kumar et al., 2024; Yong et al., 2018). The risk of infection changes over time, with most opportunistic infections typically occurring within 1 year after transplantation (Wang et al., 2024). A recent study showed that the incidence rate of opportunistic infections in the first year of post-transplantation was 10 times higher than in subsequent years (Pfirmann et al., 2024). Therefore, we chose CMV, EBV, and other infections within 1 year after LT as the outcomes to explore their relationship with IPV.

When calculating TAC-IPV in patients, the timing of TAC trough concentrations and the inclusion and exclusion criteria are important factors to consider. In the early stage after LT, a period of adjustment is required to stabilize the dosage of TAC as its pharmacokinetics can change due to the combination of drugs (including hydrocortisone and antibiotics). In addition, during hospitalization, patients’ TAC trough concentrations may change due to drug dose adjustment, drug combination, or bioavailability changes, which may lead to an overestimation of daily variability (Shuker et al., 2015). Most of the studies calculated IPV using TAC trough concentrations from 6 months after transplantation. In order to avoid the overlap between IPV calculation period and the outcomes, we collected the TAC trough concentrations from 4 to 9 months post-transplant; however, this approach showed limited effect. In order to avoid the bias caused by the exclusion of some patients with repeated or chronic hospitalizations, and considering that patients would routinely monitor TAC trough concentrations before medication adjustment on the first day of admission, TAC trough concentration on the first day of admission was also included in the analysis. Finally, a total of 116 patients with 746 TAC trough concentrations were included. The patients were grouped by the median TAC-IPV (32.31%) for comparison.

### 4.2 Influencing factors of TAC-IPV

TAC is mainly metabolized by cytochrome P450 (CYP450), so the inhibitors and inducers of CYP3A4 and/or P-glycoprotein can change its blood concentrations and bioavailability (Elens et al., 2011; Brunet et al., 2019). In our study, we found that, in addition to the effects of drug combinations reported in the literature on TAC trough concentration, drug combination is also associated with high IPV. We suspect that the simultaneous use of these drugs along with TAC, without concurrent dose adjustments, may lead to significant fluctuations in blood concentration, thereby increasing TAC-IPV. Despite this, during TAC treatment, drugs or foods that can change the blood concentration of TAC should be used with caution, and the calculation of IPV should be included in the follow-up period; this may help stabilize TAC-IPV in patients and improve prognosis.

In plasma, 75%–99% of TAC is highly bound to albumin, a1-acid glycoprotein, and RBC, with 85%–98% bound to RBC (Staatz and Tett, 2004; Piletta-Zanin et al., 2021; Wong, 2001). In clinical practice, TAC is usually measured by the total concentration in whole blood, so the whole blood concentration will be proportional to the RBC (Størset et al., 2014); in other words, low HCT levels may lead to a decrease in whole blood concentration but do not affect overall plasma TAC exposure (Koomen et al., 2023; Piletta-Zanin et al., 2021). It seems that TAC blood concentrations may be overestimated when HCT levels are low.

### 4.3 TAC-IPV and EBV infection within 1 year

In adult organ transplantation, studies on TAC-IPV have suggested that high-IPV level was a risk factor for post-transplant diabetes mellitus, CMV infection, GVHD, hepatocellular carcinoma recurrence, and other complications (Lucino et al., 2022; Jeong et al., 2022). However, it is unclear whether this is also the same case in children as in adults as the reports on TAC-IPV after pediatric LT are deficient. In our study, a small number of patients developed diabetes, hypertension, and GVHD within 1 year after LT, so statistical differences could not be compared at this time. We found that the incidence rate of EBV infection was higher in the high-IPV group than in the low-IPV group, but no statistical difference (*p* = 0.272) was observed. A recent study of 1 year prognosis after pediatric LT showed similar results, with no difference in incidence between the high- and low-IPV (CV < 45%) groups (*p* = 0.812) (Chen et al., 2024). Although the timing and grouping of IPV were inconsistent, we found the same results, leading us to suspect that TAC-IPV may not be associated with EBV infection in pediatric LT patients, and the prediction of EBV infection using IPV may not be ideal. Recent studies have found differences in the gut microbiome and nutritional status between children infected with EBV and those uninfected, which provides new hope for further research (Wang et al., 2023; Zhou et al., 2024).

### 4.4 TAC-IPV and CMV infection within 1 year

CMV is one of the most common opportunistic infections after pediatric LT, but the relationship between TAC-IPV and CMV infection after transplantation remains unclear. Previously, in kidney transplantation, studies have shown that high IPV is correlated with CMV viremia (Choi et al., 2022). In pediatric LT, two studies found no association between high IPV and CMV viremia within 1 year or late infection (Chen et al., 2024; van der Veer et al., 2019). Defrancq et al. (2019) found that within 1 year after LT in children, high IPV was correlated with CMV infection, but this result was not significant (*p* = 0.07). In our study, both correlation and Kaplan–Meier analysis showed that a strong association between high IPV and CMV infection (*p* < 0.001; H R = 2.34 [1.45; 3.76], *p* < 0.001) in children within 1 year after LT. The differences in the timing of IPV calculation and the frequency of CMV monitoring may explain the different results of the studies. Interestingly, in our study, there was no statistically significant difference in the mean TAC trough concentration between CMV-infected and uninfected patients (*p* = 0.903). This may suggest that TAC-IPV has a stronger predictive potential for CMV infection than TAC trough concentration in the early post-transplant.

### 4.5 TAC-IPV and transplant rejection within 1 year

In 2010, Pollock-BarZiv SM, in a study of 144 children with organ transplantation, found for the first time that the fluctuating TAC concentration was an independent risk factor for delayed acute rejection after transplantation (OR = 1.6)(Pollock-Barziv et al., 2010). Natalia Riva (Riva et al., 2018) retrospectively studied 72 pediatric LT patients and found that high IPV was a risk factor for post-operative acute rejection. On this basis, we conducted a simple survival analysis and found that, after taking time into account, there was still a significant difference in incidence between the two groups, high IPV (CV > 32.31%) was significantly associated with the occurrence of transplant rejection within 1 year (HR = 3.17 [1.47; 6.86], *p* = 0.005).

### 4.6 TAC-IPV and readmission due to infection within 1 year

We have also observed that pediatric patients after LT are often rehospitalized repeatedly or chronically due to various infections. Previous studies on infection mostly focused on CMV, EBV, and other specific pathogens, with less attention paid to mild infections caused by common bacteria, fungi, or unknown pathogens in children after LT. Although these infections rarely cause serious consequences, they can lead to repeated hospitalization and can seriously affect the quality of life of children and their families. Therefore, we also collected the frequency and related information of patients readmitted due to infection. Finally, 140 instances of readmission due to infection within 1 year after LT were collected. There were 45 times in the low-IPV group, and 56.9% (33 cases) of patients were not readmitted to the hospital due to infection within 1 year after LT. There were 95 times in the high-IPV group, with the highest number of readmissions for infections being six times in 1 year. We found significant differences between the two groups, the frequency of readmission for infection was significantly higher in the high-IPV group than in the low-IPV group (*p* = 0.003), and the distribution of readmissions due to infection was uniform over the 1-year post-transplant period. This indicates that patients with high IPV have a higher risk of experiencing more severe and frequently infections. Some children may also miss school due to repeated hospitalizations.

In our study, as in other studies, the outcome and the trough concentration of TAC coincided, so the causal relationship between the two needs to be interpreted with caution. However, it is undeniable that there is a correlation between IPV and CMV infection, which suggests that IPV has the potential to be an important and effective predictor for building predictive models, particularly if the two are separated in time. This is the next step in our research.

## 5 Conclusion

In summary, there was no statistical difference between high- and low-IPV groups for EBV infection. However, for post-transplant rejection, CMV infection, and rehospitalization due to infection after LT, IPV is an important potential predictor, especially for CMV infection, where it is superior to TAC trough concentration in the early post-transplant period. It is recommended that IPV calculation be included in the follow-up period. Hematocrit and concomitant medications were independent influencing factors for TAC-IPV (*p* < 0.05). We recommend that drugs that can change the concentration of TAC should be used cautiously during treatment. When adjusting the medication regimen, the trough concentration of TAC should be monitored regularly, and the IPV of patients should be calculated to avoid drastic adjustments. Patients with high IPV should receive intensive care. Increasing patient compliance, reminding the children and their families to pay attention to infection prevention, avoiding contact with infectious sources, and closely monitoring the occurrence of adverse reactions can help provide guarantee for safe and rational drug use and improve the quality of life of patients after LT.

## Data Availability

The raw data supporting the conclusions of this article will be made available by the authors, without undue reservation.
